# Recent progress on covalent organic framework materials as CO_2_ reduction electrocatalysts

**DOI:** 10.3389/fchem.2022.942492

**Published:** 2022-07-22

**Authors:** Yang Fan, Mengyin Chen, Naizhang Xu, Kaiqiang Wang, Qiang Gao, Jing Liang, Yubing Liu

**Affiliations:** ^1^ Jiangsu Engineering and Technology Research Center of VOCs Treatment, Environmental Engineering College, Nanjing Polytechnic Institute, Nanjing, JS, China; ^2^ International Collaborative Laboratory of 2D Materials for Optoelectronics Science and Technology of Ministry of Education, Institute of Microscale Optoelectronics, Shenzhen University, Shenzhen, China; ^3^ Key Laboratory of Mesoscopic Chemistry of MOE, Jiangsu Provincial Key Laboratory of Vehicle Emissions Control, School of Chemistry and Chemical Engineering, Nanjing University, Nanjing, China

**Keywords:** CO_2_ reduction, electrocatalysts, carbon neutralization, sustainability, COFs

## Abstract

CO_2_ emission caused by fuel combustion and human activity has caused severe climate change and other subsequent pollutions around the world. Carbon neutralization *via* various novel technologies to alleviate the CO_2_ level in the atmosphere has thus become one of the major topics in modern research field. These advanced technologies cover CO_2_ capture, storage and conversion, *etc*., and electrocatalytic CO_2_ reduction reaction (CO_2_RR) by heterogeneous catalysts is among the most promising methods since it could utilize renewable energy and generate valuable fuels and chemicals. Covalent organic frameworks (COFs) represent crystalline organic polymers with highly rigid, conjugated structures and tunable porosity, which exhibit significant potential as heterogeneous electrocatalysts for CO_2_RR. This review briefly introduces related pioneering works in COF-based materials for electrocatalytic CO_2_RR in recent years and provides a basis for future design and synthesis of highly active and selective COF-based electrocatalysts in this direction.

## Introduction

In modern societies, human activities and the development of industries primarily depend on fossil fuel combustion as energy source. One severe consequence of excessive consumption of fossil fuels is the considerable large amount of CO_2_ emission which is the major cause of greenhouse effect, climate change, and many other subsequent worldwide pollution issues (Bauer et al., 2016). It was reported that the global CO_2_ concentration in the atmosphere has increased from 353 to 409 ppm in the past 30 years and is predicted to reach over 600 ppm within this century (Mikkelsen et al., 2010; Yang et al., 2020).

To meet the demand of carbon neutralization and carbon recycling, a myriad of sustainable technologies have been developed to mitigate the level of atmospheric CO_2_, including CO_2_ capture, storage, and transferring CO_2_ into other useful carbonaceous molecules (Aresta et al., 2014; Yi et al., 2015; Liu et al., 2016; Abanades et al., 2017; Majumdar and deutch, 2018; Zheng et al., 2018). Photocatalysis, electrocatalysis, biological transformations, chemical fixation, and hydrogenation of CO_2_ have been extensively developed in the recent decades (Halmann, 1978; Tu et al., 2014; Duan et al., 2017; Kattel et al., 2017; Nguyen et al., 2018).

Among these, electrocatalytic CO_2_ reduction reaction (CO_2_RR) has been regarded as one of the most attractive methods to address the issue of renewable utilization of CO_2_ (Kuhl et al., 2012; Kondratenko et al., 2013; Al-Omari et al., 2018; Bushuyev et al., 2018; Chen et al., 2018; Tackett et al., 2019; Zhang M.-D. et al., 2020; Franco et al., 2020; Sun et al., 2020; Yang et al., 2020; Liang et al., 2021). This could be attributed to many reasons. First, given the advantages of electrocatalysis, the ease of controllability of the reaction rate and selectivity *via* applied potential current density makes CO_2_RR possible to be carried out under ambient conditions. Second, pure or a mixture of valuable C_1_/C_2_ carbon feedstocks such as CO, CH_4_, formic acid, or ethylene could be generated directly from electrochemical CO_2_RR as the main products *via* some multi-electron multi-proton pathways (Liu et al., 2015). Moreover, utilization of renewable energy (solar and windy) as the electricity source could fulfill the carbon cycle without emitting additional CO_2_ (Kumar et al., 2016). In addition, it is also feasible to scale up the electrochemical reaction system for mass productions (Qiao et al., 2014).

Despite these advantages, electrocatalytic CO_2_RR still suffers several technological obstacles, limiting the pace of its further development (Pletcher, 2018; Ross et al., 2019). The high C=O bond energy (805 kJ mol^−1^) makes the CO_2_ molecules chemically inert and thermodynamically stable (Glockler, 1958; Gottle and Koper, 2017). Thus, large activation energy is required to cleave the C=O bond with one electron and reorganize the linear molecule to its bent form in the electrocatalytic CO_2_RR process (Wang et al., 2018b). As a result, a highly negative reduction potential of −1.90 V (vs. SHE) is required for a single-electron reduction of CO_2_ on a non-catalytic electrode (Schwarz and Dodson, 1989). In addition, selectivity of products is another challenge of electrocatalytic CO_2_RR (Li et al., 2018; Zheng et al., 2019). Since it is usually carried out in aqueous protic media, a severe hydrogen evolution reaction (HER) at 0 V (vs. SHE) and some other side reactions will strongly interfere with the selected product formation and lower the Faraday efficiency (FE) (Zhou and Sun, 2017). To overcome these drawbacks, the creative design and synthesis of electrocatalysts with defined and stable structures along with excellent catalytic activity, product selectivity, and long-term durability are in urgent demand.

Covalent organic frameworks (COFs) have emerged as a novel type of porous crystalline polymer materials devised for various functions (Côté et al., 2005; Feng et al., 2012; Ding and Wang, 2013; Huang et al., 2016; Segura et al., 2016; Bisbey and Dichtel, 2017; Lohse and Bein, 2018; Chen et al., 2020; Geng et al., 2020). This type of reticular porous covalent frameworks is typically constructed by various organic building blocks consisting of only light nonmetal elements such as carbon, nitrogen, boron, and oxygen, which are uniformly linked by covalent bonds (Uribe-Romo et al., 2009). Compared to other porous polymer materials, COFs possess many unique features. Depending on the spatial geometric structures and functionalities of the building blocks and linkage molecules, the frameworks display rigid and highly ordered structures with 2D layer or 3D network topologies. COFs also have tunable porosity, which renders them extremely large accessible surface area and abundant active sites. Given these extraordinary characteristics, the COF-based materials have found wide applications in myriads of applications such as gas storage and separation, catalysis, photovoltaics, optoelectronics, drug delivery, and energy storage since the first report by Yaghi’s group in 2005.

In addition, since most of the COFs are synthesized *via* cross-coupling or condensation reactions between organic aromatic building blocks and linkers, the whole framework has large conjugation structures and shows semiconducting properties with tunable bandgaps, which renders them good platforms for electrocatalysis (Dogru and Bein, 2014; Lin and Chen, 2018; Lin et al., 2018; Song et al., 2019; Cui et al., 2020; Sharma et al., 2020; Guan et al., 2021; Zhao et al., 2021; Lee et al., 2022). To further improve the conductivity, COFs could be hybridized with other conductive materials such as graphene or carbon nanotubes, and the incorporation of transition metal active sites further enhances the catalytic activity and selectivity due to synergic effect between metals and COF supports. Indeed, recent years have witnessed much progress in the design and synthesis of COF-based materials for various electrocatalytic reactions including oxygen reduction and evolution reactions (ORR and OER), hydrogen evolution reaction (HER), and especially CO_2_ reduction (CO_2_RR) for the intention of energy conversion and fuel generation. Moreover, the large and tunable porous structures of COFs promote the diffusion and adsorption of CO_2_, and desorption of the resulting products, making them ideal electrocatalysts for CO_2_RR.

Despite the current progress, the development of COF-based electrocatalysts for CO_2_RR is still in its infancy stage. Herein, we will briefly review some of the seminal and representative work in this field. Although several previous reviews have come out dealing with the electrocatalytic CO_2_RR by COFs, focus in this work will be on the ingenuity of the design of electrocatalytic COF-based materials, and discussions will be divided by the types of high-value fuel products. In the end, we will also suggest the current challenges associated with and direction for future development of highly efficient COF-based electrocatalysts toward CO_2_ conversion.

## CO_2_ reduction to CO

From a mechanistic perspective, the CO_2_RR theoretically involves one-, two-, four-, six-, or eight-electron reduction pathways. However, the most common product described in practical experiments in the mainstream studies of electrochemical CO_2_RR is CO, which is the simplest form of the reduction product *via* a two-electron transfer pathway.

**FIGURE 1:**
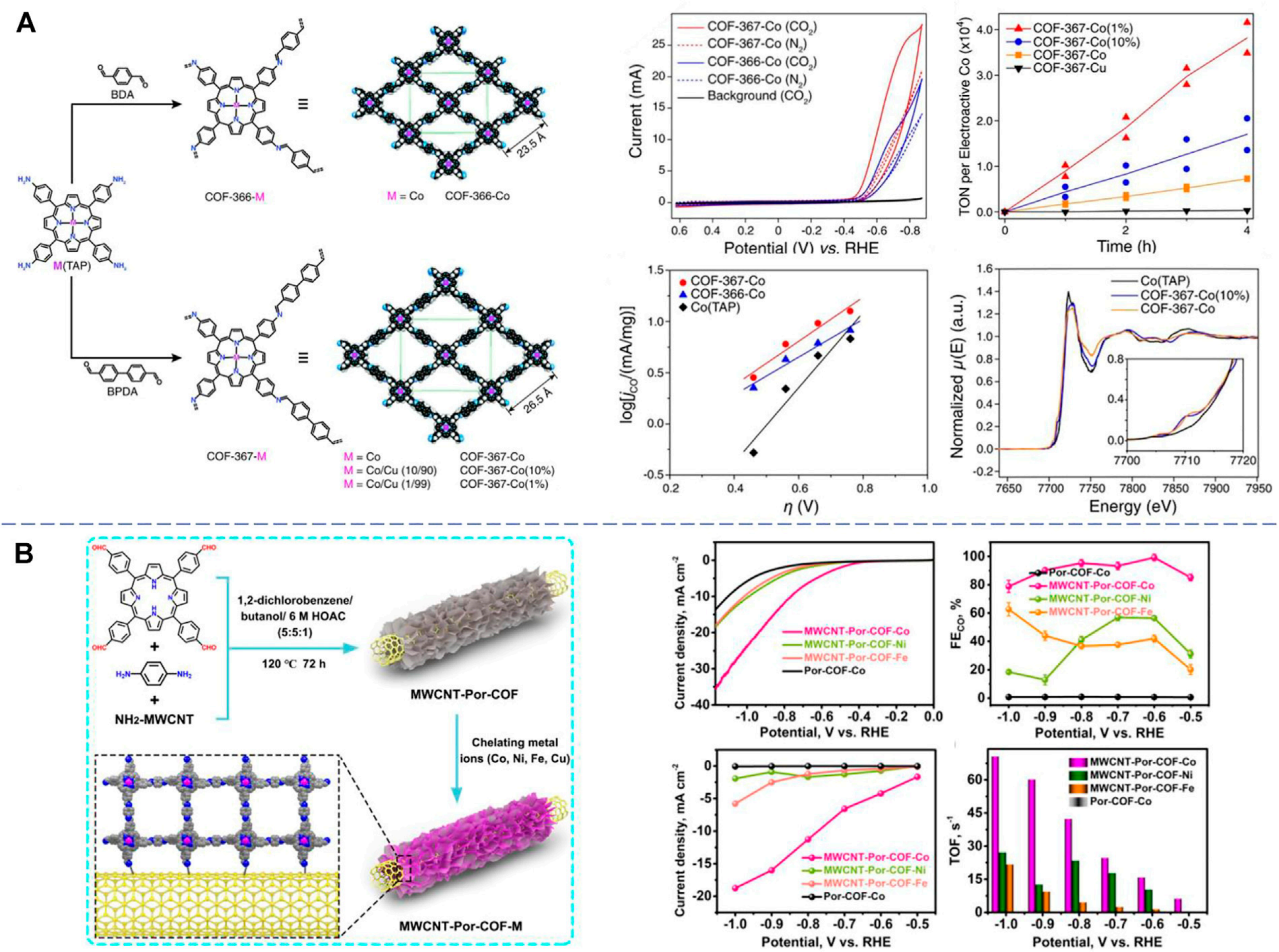
**(A)** Structures, electrochemical characterizations, and carbon dioxide reduction performance of the metalloporphyrin-derived 2D covalent organic frameworks COF-366-Co and COF-367-Co [reproduced from Lin et al. (2015) with the permission of AAAS]. **(B)** Design and synthesis of MWCNT-Por-COF-M and its electrochemical characterizations and carbon dioxide reduction performance [reproduced from Dong et al. (2022) with the permission of Elsevier].

In 2015, Yaghi et al. first reported the modular optimization of covalent organic frameworks for the electrochemical reduction of CO_2_ to CO (Lin et al., 2015). In this pioneering work, the authors synthesized two types of COFs (COF-366-Co and COF-367-Co) by reacting a molecular cobalt catalyst 5,10,15, and 20-tetrakis(4-aminophenyl)porphinato]cobalt Co(TAP) with two dicarboxaldehydes BDA and BPDA *via* imine condensation (Figure 1A). Characterizations demonstrated a stacking 2D sheet configuration consisting of 1D channels. In terms of electrocatalytic activities of CO_2_ reduction to CO, the catalysts exhibited high Faraday efficiency over 90% and turnover frequencies of 9,400 hour^−1^ at the overpotential of −0.55 V in the neutral aqueous condition. This activity was a 26-fold improvement compared to the sole molecular cobalt catalyst, and the COF catalysts could stand at least 24 h. The activity enhancement was attributed to the electronic structure of cobalt catalytic centers within the framework as revealed by X-ray absorption. In a later work by the same group, the electronic properties of 2D framework COF-366-Co were tuned by introducing electron-donating (-OMe) or electron-withdrawing (-F) groups on the BDA linker motif in the parent framework (Diercks et al., 2018). Electrocatalytic measurements showed that both catalytic activity and selectivity are further improved with the optimized 366-F-Co catalyst. X-ray absorption measurement revealed the synergetic effect between the inductive functionalities and the metal catalytic centers. Deng et al. further developed both 2D and 3D reticular COF-300-AR and COF-366-M-AR by directly reducing the corresponding parent COFs COF-300 (a previously reported COF synthesized with tetra(4-aminophenyl)methane (TAPM) and BDA *via* imine condensation) and COF-366-M with amine linkages (Liu et al., 2018). The amino groups turned out to improve both the crystallinity and chemical stability of COFs in acidic or basic environment, and FEs of CO conversion are 53% at −0.70 V and 80% at −0.85 V. Mechanistic studies showed the existence of carbamate intermediates facilitated by the amino groups during the activation of CO_2_. These consecutive works established the foundation of COF electrocatalysts for CO_2_RR.

COFs with phthalocyanine (Pc) centers often enable excellent electron transfer in the framework. Gao et al. first developed a series of cobalt-COFs constituted by molecular cobalt–phthalocyanine and boronic acid linkers *via* esterification reactions (Yao et al., 2018). Owing to the integration of storage function of the optimized Co-Pc-PBBA, the electrocatalytic reduction was carried out in the CO_2_ gas phase, which led to a nearly 100-fold CO_2_ concentration compared to the normal aqueous phase. The overpotential was considerably lowered, and this brought rate enhancement of CO production. Jiang et al. later synthesized a similar COF with cobalt phthalocyanine center and tetraone linkage *via* imine condensation (Huang et al., 2020). As a result, the whole 2D lattice exhibited full π conjugation along both *x* and *y* axes. The obtained Co-Pc-PDQ showed a Faraday efficiency of over 96% with an exceptional long-term turnover frequency of over 11,400 h^−1^, giving a 32-fold improvement over molecular catalysts. A similar Ni-Pc-PT conductive COF 2D nanosheet later developed by Cao et al. also provided over 93% high CO selectivity under a wide overpotential range of −0.60 to −1.10 V with 35 mA cm^−1^ current density in aqueous solution (Zhang M.-D. et al., 2020). Lan et al. designed and synthesized a series of COFs from cobalt and nickel phthalocyanine and tetrafluorophthalonitrile *via* dioxin linkers to improve stability (Lu et al., 2021). Outstanding Faraday efficiencies of over 96% were achieved in the electrocatalytic reduction of CO_2_ to CO, and when the system is coupled with photo irradiation, both the FE and current density were further improved. Two-dimensional polyimide-linked phthalocyanine COFs CoPc-PI-COF-1 and CoPc-PI-COF-2 developed by Jiang et al. showed great stability in strong acid aqueous solution and 87%–97% FE at the overpotentials between −0.60 and −0.90 V in the basic aqueous phase (Han et al., 2021).

The covalent triazine framework (CTF) represents a specific class of COF constitution. The building block molecules are connected by the cyano groups on their structures forming six-membered triazine rings, and in this case, no other reactants or linkers are necessary. Zhuang et al. developed a Ni porphyrin-based covalent triazine framework (NiPor-CTF) for electrocatalytic CO_2_ reduction (Lu et al., 2019). Still, remarkable results were obtained with an extremely high current density of 52.9 mA cm^−1^. Density function theory calculation suggested the key step of *CO_2_ transition to *COOH on the atomically dispersed NiN_4_ active centers. A rationally designed covalent triazine framework CTF-B featuring CuN_2_Cl_2_ active sites not only can convert CO_2_ to CO but also exhibits electrocatalytic selectivity for C_2_ products such as acidic acid and ethylene with a FE of 68.4% in total (Ma et al., 2020).

Sulfur-containing aldehyde linker molecules such as tetrathiafulvalene (TTF) and thieno[3,2-b]thiophene-2,5-dicarbaldehyde (TT) were also exploited to constitute COFs with porphyrins to further improve the conductivity. These sulfur-containing linkers could serve as electron carriers and form oriented electron transfer pathways with metalloporphyrin active sites during the electrocatalytic process. Related works by Lan et al. and Cao et al., respectively, demonstrate Co TT or TTF COFs giving exceptional results, paving a new way for constructing novel COFs for electrocatalysis (Zhu et al., 2020; Wu et al., 2021).

In addition to these, several other COFs for the highly efficient electrocatalytic CO_2_ reduction to CO in recent years were also worth mentioning. These unique structures include a single Mn atom loaded on bipyridyl-based COFs (COF_bpyMn_) *via* post-modification (Dubed Bandomo et al., 2021), a crown ether B18C6 embedded Co(TAPP)-COF (An et al., 2021), and a zwitterionic ultrathin cobalt tetraamino phthalocyanine-squaraine based COFs (COP-SA) (Song et al., 2021). All these ingenuous works guide the rational design of novel COF electrocatalysts for CO production from CO_2_.

## CO_2_ reduction to hydrocarbon fuels

Although electrochemical CO_2_ reduction reaction catalyzed by a variety of conductive COF-based materials has been intensively investigated and remarkable results have been achieved during the past decade, an obvious bottleneck still exists in the type of reaction. As mentioned in the previous section, most of the COF-based catalysts direct the reaction to produce two-electron transferred product CO which is generated almost exclusively during the reaction. In these cases, the reduction of CO_2_ stays at the stage of CO at the active sites of COF catalysts, especially those with the metalloporphyrin active centers. However, hydrocarbon fuels with higher added value such as CH_4_, C_2_H_4_, and other deeply reduced multicarbon (C_2+_) products generated in CO_2_RR by COFs were barely reported. From a mechanistic viewpoint, the generation of these hydrocarbon fuels requires multiple electron (>2) transfer, and this process should be coupled with proton transfer. Thus, innovative COF catalysts for electrochemical CO_2_RR that are designed to meet these requirements remain a major challenge in this field.

A seminal work by Wen et al. first reported a metal-free perfluorinated covalent triazine framework (CTF)-based electrocatalysts FN-CTF-400 for highly selective CO_2_ conversion to CH_4_ with a 99.3% Faradaic efficiency in aqueous condition (Wang et al., 2018). The covalent triazine frameworks are featured in their ultralarge surface area and readily accessible of tuning surface functionalities by introducing heteroatom doping. Density functional theory calculations demonstrated that the high selectivity of CH_4_ production in the electrochemical CO_2_RR was probably attributed to the synergistic effect of doped F and N atoms in FN-CTF-400. Later, Huang and Cao et al. also devised copper nanoparticles (Cu NPs) immobilized on the imidazolium-functionalized covalent triazine framework (Cu/ICTF) to produce C_2_H_4_ in electrochemical CO_2_RR since Cu-based catalysts have been often utilized for generating multiple electron transfer C_2+_ products with relatively high selectivity (Mao et al., 2020). The imidazolium groups in ICTF could both improve the capture and activation of CO_2_ and thus lower the reaction energy barrier. Moreover, the Cu NPs are covalently linked and stabilized on the *in situ* formed N-heterocyclic carbenes (NHC) to form atomically dispersed active centers to prevent their deactivation. Cu/ICTF demonstrated a 35% high selectivity of C_2_H_4_ in CO_2_ electrochemical reduction along with a large current density of 4.14 mA cm^−2^ and an exceptional stability over 10 h.

In addition to the self-supported CTF, a series of well-defined nanostructures were also applied as supports of transition metal COF-based CO_2_RR electrocatalysts. Carbon nanotubes (CNTs) vertically connected with porphyrin-based covalent organic frameworks (Por-COF) nanosheets by covalent bonds have been developed for electrocatalytic CO_2_ reduction reaction to achieve CH_4_ (Dong et al., 2022). In this case, the CNT functions as both carriers to uniformly disperse Por-COF and controllers to facilitate the electron transfer pathway between porphyrin planes and metal active centers. As results, the MWCNT-Por-COF-Cu displayed high selectivity for CH_4_ with a FE of 71.2% under a flow-cell condition, which could be attributed to the *in situ* generated Cu-based nanoclusters during the electrocatalytic process. Meanwhile, the MWCNT-Por-COF-Co exhibited a remarkable catalytic activity with a FE high up to 99.3% at −0.60 V for CO production, a current density of 18.77 mA cm^−2^, and TOF up to 70.6 s^−1^ (Figure 1B). Lan et al. also designed tunable 1D nanofibers (NFs) and hollow tubes (HTs) as COF materials (Liu et al., 2021). These anthraquinone-based (AAn) COFs with superstructures could be post-modified with various transition metals for CH_4_ production, and the obtained AAn-COF-Cu (NF) and OH-AAn-COF-Cu (HT) exhibit exceptional Faraday efficiencies of 77% (128.1 mA cm^−2^, 0.90 V) and 61% (99.5 mA cm^−2^, 1.00 V), under flow-cell condition, respectively, the former of which is the highest among all the reported COF catalysts for CO_2_RR.

Carbon materials such as graphene oxide (GO) were also applied as linker molecules for building the 3D graphene framework (CGF) because of its easy accessibility, good conductivity, and functionalities. The CGF-based cobalt porphyrin catalysts developed by Bettelheim et al. exhibited a FE of about 20% for CH_4_ in CO_2_RR due to the strong irreversible adsorption of CO_2_ and stabilization of a key intermediate afforded by the free amine groups on the graphene (Bochlin et al., 2021).

## Summary and perspectives

Electrocatalytic CO_2_ reduction reaction is currently regarded as one of the most efficient and straightforward technologies for alleviating the CO_2_ level in the atmosphere utilizing renewable energy and closing carbon cycle. Although various homogeneous and heterogeneous catalysts have been invented to improve the catalytic activity and product selectivity in electrocatalytic CO_2_RR, challenges associated with these catalysts also exist for the COF-based electrocatalysts. Therefore, this promising research field still awaits further development, and future devotion in this direction should continue to focus on improving the catalytic activities and product selectivities under various reaction conditions.

**FIGURE 2:**
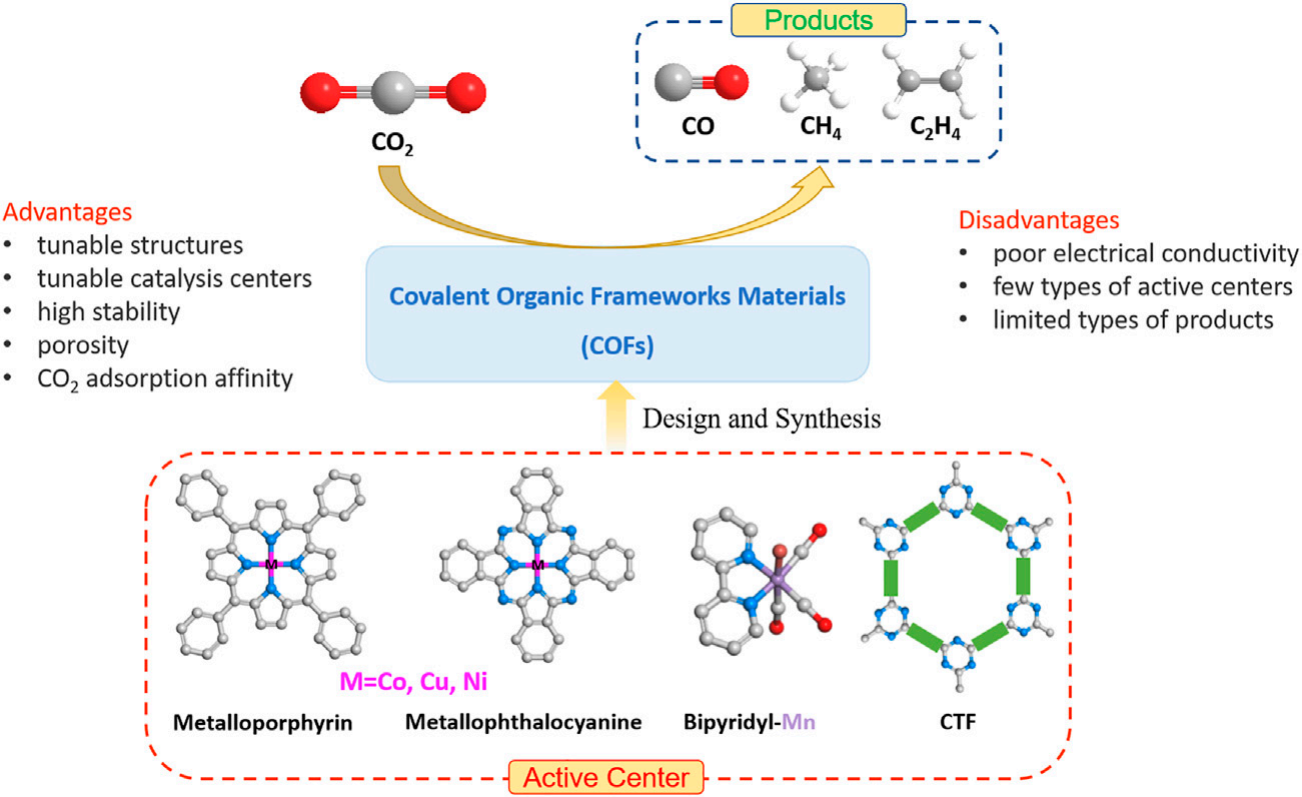
COF-based electrocatalysts for CO_2_RR.

To be more specific, given the studies in recent years, it is obvious that the COF-based electrocatalysts for CO_2_RR still have problems such as poor electrical conductivity, few types of active centers, and the target product is mainly CO (Figure 2). Although the conductivity of electrocatalysts can be improved by compounding COFs with conductive materials such as graphene and CNT, we feel that the investigation of COF materials with inherent high conductivity for CO_2_RR can be more interesting. In addition, it is worth noting that most of the active centers of the COF-based electrocatalysts are in M-N_4_ structures (such as metalloporphyrin and metallophthalocyanine), which have been widely considered to exhibit excellent catalytic performance in electrocatalytic reactions. Exploring other types of active centers with different metal centers and building blocks would be another potential direction. Furthermore, in the electrocatalytic CO_2_RR by COF-based electrocatalysts, the obtained target product is primarily CO, while the high value-added hydrocarbons are more difficult to be obtained. Future studies looking deeper into the CO_2_RR mechanism may be conducive to the development of efficient catalysts for the production of hydrocarbon products. Last, since the COF-based electrocatalysts have demonstrated excellent performance toward CO_2_ adsorption and conversion, one step further could be taken by considering how to apply these COF electrocatalysts in organic synthesis exploiting CO_2_ *via* its reduction and insertion, and produce more high-value fine chemicals. We hope this review could offer new insights in the field of electrocatalytic CO_2_RR and pave ways for the design of more innovative COF-based electrocatalysts in the future.

## Data Availability

The original contributions presented in the study are included in the article/Supplementary Material. Further inquiries can be directed to the corresponding author.
